# Efficacy of Fuzhiqing ointment in mild-to-moderate atopic dermatitis: protocol for a multicenter, randomized, double-blind, placebo-controlled trial

**DOI:** 10.3389/fmed.2025.1686208

**Published:** 2026-01-12

**Authors:** Xiangjin Gao, Zhen Duan, Xiuli Xiao, Fanlingzi Shen, Ruiqi Cai, Xiuqi Zhang, Quanruo Xu, Rui Zhang, Ruiping Wang

**Affiliations:** 1 Clinical Research Center, Shanghai Skin Disease Hospital, Tongji University, Shanghai, China; 2 School of Public Health, Shanghai University of Traditional Chinese Medicine, Shanghai, China; 3 Dermatology Department, Shanghai Baoshan District Integrated Traditional Chinese and Western Medicine Hospital, Shanghai, China; 4 School of Medicine, Tongji University, Shanghai, China

**Keywords:** atopic dermatitis, traditional Chinese medicine, Fuzhiqing ointment, herbal pharmacology, randomized controlled trial, clinical trial protocol

## Abstract

**Background:**

Atopic dermatitis (AD) is a chronic inflammatory skin condition that presents a significant disease burden, being the most prevalent non-fatal skin disease globally. While topical treatments play a vital role in managing mild-to-moderate AD, existing therapies often offer limited efficacy or have undesirable side effects. Fuzhiqing ointment, formulated from 15 traditional Chinese herbs, has demonstrated promising effects in anti-inflammatory, antipruritic, and anti-infective properties. Despite its widespread use in clinical practice, particularly for treating itching skin diseases, high-quality clinical evidence supporting its effectiveness in AD remains scarce. This trial seeks to address this gap by evaluating the clinical efficacy of Fuzhiqing ointment in managing mild-to-moderate AD, providing critical evidence for its potential integration into mainstream dermatologic care.

**Objectives:**

This multicenter, randomized, double-blind, placebo-controlled clinical trial aims to assess the efficacy of Fuzhiqing ointment in alleviating the symptoms of mild-to-moderate AD. We hypothesize that the inclusion of Fuzhiqing ointment in the treatment regimen will lead to a significant improvement in clinical outcomes compared to placebo, offering an innovative therapeutic approach in the AD treatment landscape.

**Methods and analysis:**

A total of 210 patients with mild-to-moderate AD will be recruited from 10 hospitals across China between September 2025 and February 2026. Participants will be randomly assigned in a 2:1 ratio to receive either the treatment (urea vitamin E cream combined with Fuzhiqing ointment; *n* = 140) or the control (urea vitamin E cream combined with placebo Fuzhiqing ointment; *n* = 70). Both groups will apply the treatments twice daily for 2 weeks, followed by a 4-week observational follow-up period. The primary outcome will be the proportion of patients achieving ≥50% improvement in the Eczema Area and Severity Index (EASI) score at week 2 (EASI_50_). Secondary outcomes will include changes in the EASI, Numerical Rating Scale (NRS), Investigator’s Global Assessment (IGA), Dermatology Life Quality Index (DLQI), and Atopic Dermatitis Control Tool (ADCT) at weeks 1 and 2, as well as the EASI, NRS, and adverse events at week 6. Statistical analyses will be performed using SAS 9.4, with significance defined at a two-tailed *α* level of 0.05.

**Results:**

In this study, ethics approval was obtained in December 2024 and registered in Chinese Clinical Trial Registry in January 2025. Participant recruitment was commenced in February 2025 and is expected to be completed by February 2026. Data analysis will be initiated in May 2026, and the preliminary trial results are expected to be submitted for peer-reviewed publication in December 2026.

**Clinical trial registration:**

https://www.chictr.org.cn/, Identifier ChiCTR2500095971.

## Introduction

Atopic dermatitis (AD) is a chronic, recurrent, inflammatory skin disease characterized by severe itching, dry skin, and typical eczematous lesions (1). Its pathogenesis is complex, involving skin barrier dysfunction, immune dysregulation, environmental factors, and genetic susceptibility. AD is associated with a significant disease burden and represents the highest burden among all non-fatal skin diseases (2). Beyond cutaneous manifestations, AD profoundly impacts patients’ quality of life, contributing to sleep disturbances, cognitive impairments and heightened risks of anxiety and depression (3).

In recent years, numerous treatments for AD have been developed, including emollients, corticosteroids, calcineurin inhibitors, antihistamines and biologics (4–6). Topical treatment is crucial for the long-term management of AD, particularly in those with mild-to-moderate symptoms. However, current therapeutic options have limitations, including the potential side effects of long-term potent corticosteroid use, inadequate efficacy or frequent recurrence in some patients, and concerns regarding the applicability and safety of systemic therapies (7–9). Therefore, developing safe, effective, and easily manageable new topical treatment options is still important clinical need.

Traditional Chinese Medicine (TCM) has been increasingly recognized for its therapeutic potential in treating mild-to-moderate AD (10–15). One such formulation, Fuzhiqing ointment, contains a combination of 15 Chinese medicinal herbs, including Tinospora root, Rhubarb, Golden cypress, *Ardisia crenata*, Chinese violet, Wild chrysanthemum, Hemsleya amabilis, *Sophora flavescens*, Paris polyphylla, Airpotato yam, Turmeric, *Sanguisorba officinalis*, Kuding tea, Borneol, and Menthol. Each herb in the formulation plays a distinct role in addressing the underlying pathophysiology of AD. For instance, *Sophora flavescens* has known anti-inflammatory and antibacterial properties, which help reduce the inflammatory responses that characterize AD. Turmeric is recognized for its potent antioxidant and anti-inflammatory effects, supporting skin healing and reducing irritation. Other herbs, such as *Sanguisorba officinalis* and Paris polyphylla, contribute to enhancing skin barrier function by promoting moisture retention and skin regeneration. Existing research has demonstrated that Fuzhiqing ointment exerts anti-inflammatory, antipruritic, and anti-infective effects, which contribute to its therapeutic efficacy in managing AD symptoms. The ointment works by replenishing skin moisture and lipids, thereby aiding the restoration of the skin’s physical barrier. This action effectively reduces transepidermal water loss and helps maintain the skin’s physiological acid–base balance. Additionally, it inhibits pathogen proliferation, thereby decreasing the skin’s sensitivity to external stimuli such as allergens and irritants. The absence of hormonal components in the formulation also makes Fuzhiqing ointment a safer alternative for patients seeking long-term relief from AD, as it minimizes the risk of side effects typically associated with hormone-based treatments.

Although Fuzhiqing ointment is commonly used in clinical practice to treat itching skin diseases such as eczema and AD. However, there is currently a lack of high-quality clinical trial evidence to support its clinical efficacy in the treatment of mild-to-moderate AD. Therefore, we implement this multicenter, randomized, double-blind, placebo-controlled clinical trial to evaluate the efficacy of Fuzhiqing ointment in patients with mild-to-moderate AD. We hypothesize that Fuzhiqing ointment will effectively alleviate symptoms in patients with mild-to-moderate AD, and findings in this study will potentially offering a new treatment option for patients with AD patients.

## Methods and analysis

### Study design

**Figure 1:**
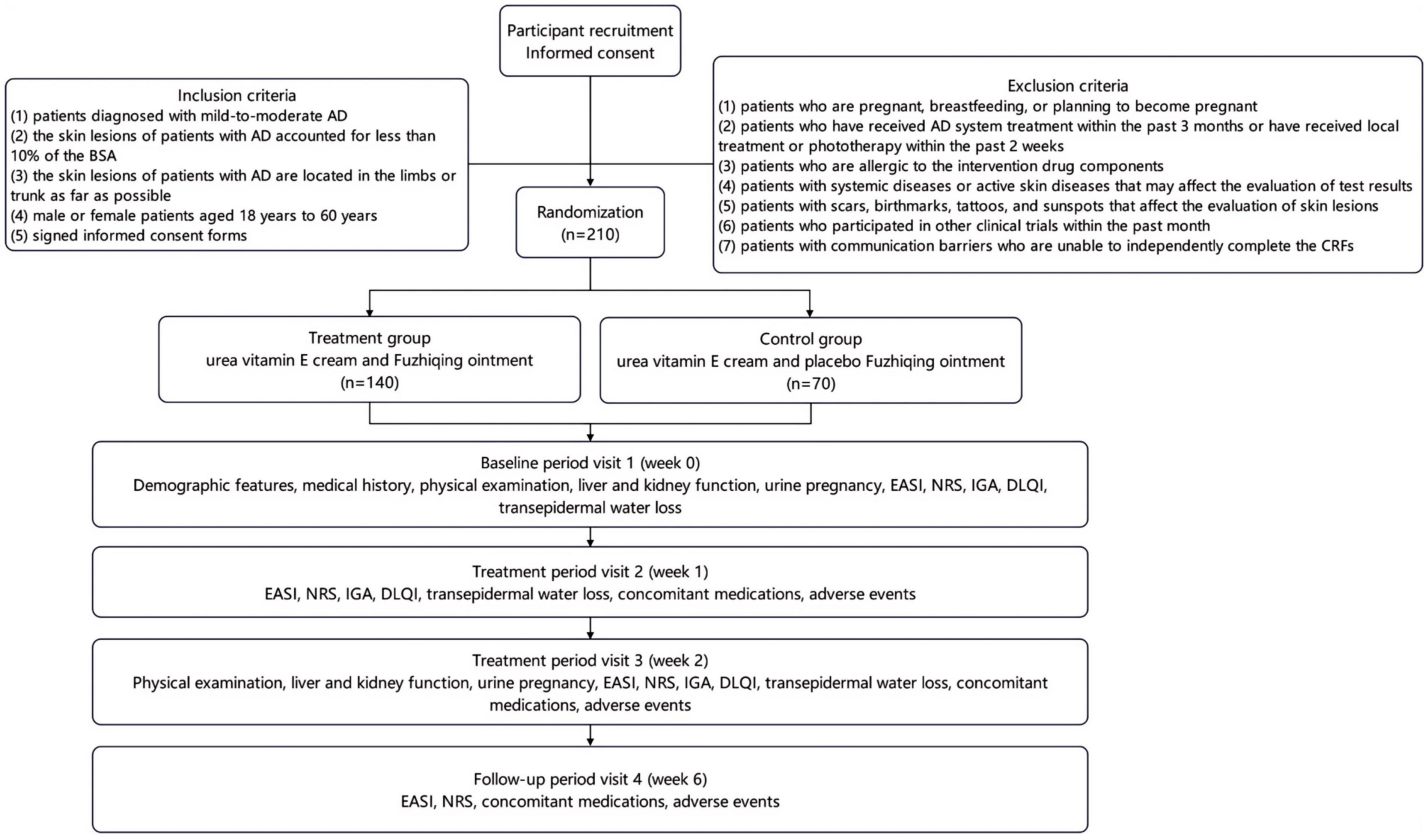
Clinical trial flowchart.

This study is a multicenter, randomized, double-blind, placebo-controlled trial to evaluate the efficacy of Fuzhiqing ointment in treating mild-to-moderate AD. The study design adheres to the SPIRIT (Standard Protocol Items: Recommendations for Interventional Trials) statement (16). A total of 210 patients with mild-to-moderate AD will be recruited in 10 hospitals between September 2025 and February 2026. Participating sites include Shanghai Skin Disease Hospital, Yueyang Hospital of Integrated Traditional Chinese and Western Medicine affiliated to Shanghai University of Traditional Chinese Medicine, China-Japan Friendship Hospital, Hangzhou Third People’s Hospital, Chongqing Traditional Chinese Medicine Hospital, The Second Affiliated Hospital of Xi’an Jiaotong University, Beijing Hospital of Traditional Chinese Medicine, Guangdong Provincial Hospital of Chinese Medicine, Peking University Third Hospital and Hubei Provincial Hospital of Traditional Chinese Medicine. A total of 210 patients with mild-to-moderate AD will be randomly allocated in a 2:1 ratio to the treatment group (urea vitamin E cream and Fuzhiqing ointment; *n* = 140) or the control group (urea vitamin E cream and placebo Fuzhiqing ointment; *n* = 70). Each patient in both groups will receive two treatment sessions per day for 2 weeks, followed by a 4-week follow-up period. The study flowchart is presented in Figure 1.

### Inclusion criteria

The inclusion criteria for mild-to-moderate AD patients include: (1) patients diagnosed with mild-to-moderate AD according to the Williams criteria [guideline for primary care of atopic dermatitis, 2022 edition (17)], (2) the skin lesions accounted for less than 10% of the body surface area (BSA), (3) the skin lesions are mainly located in the limbs or trunk, (4) male or female patients aged 18–60 years, and (5) signed informed consent forms.

### Exclusion criteria

The exclusion criteria include: (1) patients who are pregnant, breastfeeding, or planning to become pregnant; (2) patients who have received AD system treatment within the past 3 months or have received local treatment or phototherapy within the past 2 weeks; (3) patients who are allergic to the intervention drug components; (4) patients with systemic diseases or active skin diseases that may affect the evaluation; (5) patients with scars, tattoos, birthmarks, and sunspots that affect the skin lesion evaluation; (6) patients who participated in other clinical trials within a month; and (7) patients with communication barriers who are unable to independently complete the case report form (CRF).

### Sample size

The sample size was calculated based on the EASI50 achievement rate at week 2, which was defined as the proportion of patients who achieved ≥50% improvement in Eczema Area and Severity Index (EASI) score from baseline to week 2. According to previous evidence, we assume that the EASI50 achievement rate at week 2 was 60% in the treatment group and 30% in the control group (18). The sample size calculation for a 2:1 randomization design performed in PASS 15.0 (NCSS Statistical Software) indicates that 116 patients in the treatment group and 58 patients in the control group need to recruit with a 2-tailed *α* level of 0.05 and a statistical power of 80%. Considering a dropout rate of 10 and 10% multicenter effect, the final enrollment targets are 140 patients in the treatment group and 70 patients in the control group.

### Randomization

In this study, a total of 210 patients with mild-to-moderate AD will be randomly allocated to either treatment group or control group in a 2:1 ratio. We will apply cluster randomization with the combined blocks of 4 and 6 to ensure a balanced group allocation. Randomization sequences will be computer-generated by an independent statistician using SAS software (version 9.4; SAS Institute Inc). Randomization was centralized across all 10 hospitals, with 21 random numbers assigned to each hospital. Allocation concealment will be maintained through sequentially numbered, opaque envelopes securely stored with restricted access at the clinical research center of Shanghai Skin Disease Hospital.

### Blinding

In this study, both of patients with mild-to moderate AD and dermatologists (investigators) will be blinded to the treatment allocation. Furthermore, the outcome assessors, data collectors and statistician responsible for data analysis are also unaware of the group assignment during the trial period to prevent bias.

### Interventions

Patients in the treatment group will receive twice daily treatment sessions of urea vitamin E cream and Fuzhiqing ointment for 2 weeks. The detailed composition of Fuzhiqing ointment is shown in Table 1. To ensure the correct application of urea vitamin E cream and Fuzhiqing ointment, dermatologists will instruct patients to apply Fuzhiqing ointment first and urea vitamin E cream at least 5 min later to the affected area and instruct patients that one knuckle length of urea vitamin E cream and Fuzhiqing ointment should be used to cover no more than 1% of the body surface area. Moreover, the application of Fuzhiqing ointment and urea vitamin E cream should be followed by 1 to 3 min of finger massage to promote absorption.

**Table 1:** The detailed composition of Fuzhiqing ointment.

Component	Pharmacological activity
Tinospora root	antibacterial; anti-inflammatory; antiviral; anti-tumor; immunomodulatory
Rhubarb	antibacterial; anti-inflammatory; anti-tumor; hemostatic; antioxidant
Golden cypress	antibacterial; anti-inflammatory; antiviral; anti-fungal; antioxidant
*Ardisia crenata*	relieves cough and phlegm; antibacterial; anti-inflammatory; antiviral; anti-tumor; pain relief; promoting blood circulation for removing blood stasis
Chinese violet	antibacterial; anti-inflammatory; antiviral; anti-tumor; immunomodulatory; antioxidant
Wild chrysanthemum	antibacterial; anti-inflammatory; antiviral; immunomodulatory; antioxidant; cardiovascular protection
Hemsleya amabilis	antibacterial; anti-inflammatory; anti-tumor; antipyretic; analgesic
*Sophora flavescens*	antibacterial; anti-inflammatory; antiviral; anti-tumor; anti-arrhythmic; relieving asthma; anti-allergy
Paris polyphylla	antibacterial; anti-inflammatory; antiviral; anti-tumor; immunomodulatory; hemostatic; sedative; analgesic
Airpotato yam	antibacterial; antiviral; anti-tumor; anti-thyroid; hemostatic
Turmeric	anti-inflammatory; anti-tumor; antioxidant; liver and gallbladder protection; regulate blood lipids; anti-atherosclerotic
*Sanguisorba officinalis*	antibacterial; anti-inflammatory; hemostasis; antioxidant; anti-anaphylaxis; promotes wound healing and contraction; protects gastrointestinal mucosa (anti-ulcer)
Kuding tea	antibacterial; anti-inflammatory; regulate blood lipids; regulate blood pressure; antioxidant; anti-aging; anti-atherosclerotic
Borneol	antibacterial; anti-inflammatory; pain relief; local anesthesia; promote blood–brain barrier permeability; promote drug absorption
Menthol	cooling; pain relief; itch relief; promotes local blood circulation

Patients in the control group will also receive twice daily treatment sessions of urea vitamin E cream and placebo Fuzhiqing ointment for 2 weeks. The placebo Fuzhiqing ointment is made using guar gum as a thickening agent, caramel color as a coloring agent, and purified water as a filler. Therefore, it has the consistency of a traditional Chinese medicinal ointment but does not contain any active ingredients, and as such, does not have therapeutic effects. The placebo Fuzhiqing ointment is produced and provided by the same company, which maintains complete consistency with the Fuzhiqing ointment in terms of appearance, weight, color, odor, and package. The medication guidance and application of placebo Fuzhiqing ointment and urea vitamin E cream in the control group are completely consistent with that in the treatment group. In this study, no additional treatment is permitted during the study. Emergency interventions require documentation in detail and will result in the withdrawal of participation.

### Follow-up

In this study, all patients with mild-to-moderate AD will receive physical examination and skin lesion evaluation at baseline (day 0) following the enrollment, then patients will be followed up for subsequently 6 weeks and receive treatment efficacy evaluation at week 1 and week 2. The detailed information for data collection, physical examination and skin lesion evaluation is depicted in Figure 1.

### Outcomes

#### Primary outcome

The primary outcome indicator in this study is the EASI50 achievement rate at week 2. The EASI50 achievement rate is defined as the proportion of patients with mild-to-moderate AD who achieved ≥50% improvement in EASI score from baseline to week 2.

The EASI score includes the affected body surface area score (A) and clinical features score (B) of the head and neck, trunk, upper limbs, and lower limbs (19). (A) involves evaluating the percentage of affected body surface area (BSA) for each part. The percentage of affected BSA for each region is scored on a scale of 0–6: 0 (0%), 1 (<10%), 2 (10–19%), 3 (20–49%), 4 (50–69%), 5 (70–89%), and 6 (90–100%). (B) assesses four key clinical features, including erythema (E), edema/papules/infiltration (I), excoriation (Ex), and lichenification (L). Each feature is scored based on severity, ranging from 0 to 3 points (0 = none, 1 = mild, 2 = moderate, 3 = severe). The EASI score is calculated by multiplying the product of (A) and (B) with regional weighted coefficient (0.1–0.4) and then summing across all regions. The total score of EASI ranges from 0 to 72 points, with higher scores indicating greater severity. According to the EASI score, the skin lesion condition of AD patient is considered as mild (0–7 points), moderate (7–21 points), or severe (>21 points). In this study, the EASI score will be estimated at baseline (week 0), week 1, week 2, and week 6.

#### Secondary outcomes

The secondary outcome indicators include the EASI and Numerical Rating Scale (NRS) evaluated at week 1, 2, and 6; Investigator’s Global Assessment (IGA), Dermatology Life Quality Index (DLQI) and Atopic Dermatitis Control Tool (ADCT) evaluated at weeks 1 and 2 (20, 21). The comprehensive study schedule is detailed in Table 2, demonstrating the specific evaluation time-point for each indicator.

**Table 2:** Study schedule of the Fuzhiqing ointment clinical trial (6 weeks).

Indicator	Baseline	Treatment		Follow-up
	Visit 1 (week 0)	Visit 2 (week 1)	Visit 3 (week 2)	Visit 3 (week 6)
Informed consent	✓	—^a^	—	—
Demographic features	✓	—	—	—
Medical history	✓	—	—	—
Physical examination	✓	—	—	—
Blood biochemistry	✓	—	✓	—
Urine pregnancy	✓	—	✓	—
Inclusion/exclusion criteria check	✓	—	—	—
Randomization	✓	—	—	—
EASI^b^	✓	✓	✓	✓
NRS^c^	✓	✓	✓	✓
IGA^d^	✓	✓	✓	—
DLQI^e^	✓	✓	✓	—
ADCT^f^	✓	✓	✓	—
Concomitant medications	—	✓	✓	✓
Adverse events	—	✓	✓	✓

a Not applicable.

b EASI: Eczema Area and Severity Index.

c NRS: Numerical Rating Scale.

d IGA: Investigator’s Global Assessment.

e DLQI: Dermatology Life Quality Index.

f ADCT: Atopic Dermatitis Control Tool.

### NRS

Pruritus severity in patients with mild-to-moderate AD will be assessed by the validated NRS, an 11-point patient-reported instrument (0 = no pruritus, 10 = worst imaginable pruritus) (22). At each visit at baseline, week 1, week 2, and week 6, participants will assess their peak pruritus severity over the preceding 24 h using the NRS, and the NRS scores will be categorized as: 0 (none), 1–3 (mild), 4–6 (moderate), 7–8 (severe), and 9–10 (very severe).

### IGA

IGA is a validated 6-point ordinal scale enabling rapid clinical evaluation of disease severity (23). Dermatologists assess key features of skin lesion by using the following criteria: 0 = clear (no erythema), 1 = almost clear (minimal erythema; barely perceptible edema/papules), 2 = mild (mild erythema; palpable edema/papules), 3 = moderate (moderate erythema; evident edema/papules), 4 = severe (severe erythema; marked edema/papules), and 5 = very severe (confluent erythema with edema). IGA scores will be evaluated at baseline, week 1, and 2.

### DLQI

DLQI is a validated instrument that systematically evaluates health-related quality of life impairment across six domains over the preceding week, including physiological response, psychological feeling, family, interpersonal communication, occupational restrictions, social activities, and treatment response (24). Using a 4-point severity scale (0 = none, 1 = mild, 2 = severe, 3 = very severe) for each of 10 items, the DLQI score is obtained by summing up the scores of each items, which ranges from 0 to 30, with higher score indicating greater quality of life impairment. The DLQI assessments will be conducted at baseline, week 1 and 2.

### ADCT

ADCT is an important tool for AD patients to self-evaluating their disease control status. It can assess both the severity of the disease and the quality of life of patients over the past week, achieving a comprehensive evaluation of the effectiveness of AD control. It includes overall symptom severity score, number of severe itching episodes, discomfort, sleep disorders, and the impact of AD on daily life and emotions, covering multiple dimensions of long-term control. Each item is scored on a 0–4 scale (0 = none, 1 = mild, 2 = moderate, 3 = severe, 4 = very severe), with total scores ranging from 0 to 24 points. The ADCT score below 7 indicates that the disease is under control for AD patients. It will be evaluated at baseline, week 1 and 2.

### Safety assessment

In this study, patients with mild-to-moderate AD will be informed of the risks that may arise in the study before signing the informed consent form. We define adverse events as unintended signs, symptoms, or diseases occurring after treatment that are not necessarily related to the intervention. The occurrence of any adverse events will be accurately recorded in the CRF. In this study, appropriate treatment will be provided by dermatologists when encountering adverse events, the adherence condition of AD patients and the adverse events will be recorded. Meanwhile, liver and kidney function test for all participants and urine pregnancy test only for female participants will be conducted at baseline and week 2 for safety evaluation.

### Withdraw and dropout

According to the declaration of Helsinki, all participants will be respected and can withdraw from the study at any time for any reason. Personal information of participants will be collected and kept confidential. In this study, patient can withdraw at any time, and the reason for withdrawal will be recorded in the case report form (CRF).

### Data collection and management

Trained dermatologists will collect data using standardized case report form (CRF). The CRF comprises six sections: (1) demographic feature: age, gender, and census register; (2) diseases history of AD; (3) physical examination for height, weight and body mass index (BMI); (4) EASI, NRS, IGA, DLQI, and ADCT score recorded at baseline, week 1, 2, and 6; (5) liver and kidney function test for all participants and urine pregnancy test only for female participants; (6) prior drug history, concomitant medications, and adverse events.

We will establish a dedicated Data Monitoring Committee responsible for regularly reviewing the data collected throughout the study to ensure its accuracy and completeness. Adverse events will be reported according to the predefined protocol and reviewed by independent monitoring personnel. Data monitoring will involve real-time tracking and periodic summaries to ensure the early detection of potential issues.

The database will be constructed in Epidata 3.1 software with comprehensive validation rules. Data for all 210 participants will be entered using the double data entry method by two independent operators. Subsequent consistency checks between both datasets will identify discrepancies, and all inconsistencies will be resolved against source documents (original CRF) through iterative verification until 100% concordance is achieved.

### Statistical analysis

In this study, analysis will be performed by applying SAS 9.4 statistical package. Normally distributed quantitative variables will be summarized as mean and standard deviation (SD), while those with skewed distributions will be presented as median and interquartile range (IQR). The Student *t*-test will be used for inter-group comparison of quantitative variables with normal distribution, while the Wilcoxon rank-sum test will be employed for quantitative data with skewed distribution. The repeated measurement data will be analyzed by repeated measure analysis of variance. Categorical variables will be reported as frequencies and proportions (%), with group differences assessed using chi-square tests or Fisher’s exact tests. In this study, data analysis will adhere to the intention-to-treat (ITT) principle, including all randomized patients regardless of protocol adherence or study completion (25). Missing data will be handled under the missing-at-random assumption using sequential regression multiple imputation, which imputes missing values through regression models for each variable conditional on the other variables (26). Additionally, data analysis will be conducted on both the full analysis set (FAS) and the per-protocol set (PPS), and a *p*-value less than 0.05 (two tailed) is considered as statistically significant.

## Results

In this study, ethics approval was obtained in December 2024 and registered in Chinese Clinical Trial Registry in January 2025. Participant recruitment was commenced in February 2025 and is expected to be completed by February 2026. Data analysis will initiate in May 2026, and the preliminary trial results are expected to be submitted for peer-reviewed publication in December 2026.

## Discussion

This multicenter, randomized, double-blind, placebo-controlled clinical trial represents the first investigation evaluating the efficacy of Fuzhiqing ointment in Chinese patients with mild-to-moderate AD. In this study, the urea-vitamin E cream and Fuzhiqing ointment combination is anticipated to effectively alleviate AD symptoms in mild-to-moderate cases, potentially offering a novel therapeutic approach for AD management. AD profoundly impacts the physical and mental health of patients, suffering from itching, impaired self-image, social disturbance and somnipathy for a long time. Moreover, AD imposes substantial burdens on daily academic, occupational, and personal functions, contributing to significantly elevated risks of anxiety and depression among those affected individuals (27, 28). Meanwhile, AD imposes a substantial additional physical and mental burden on adolescents, adversely affecting their learning, social development, and self-esteem formation (29, 30). For AD treatment, the anti-inflammatory agents, wet wrap therapy, systemic immunology-suppressants and short-term steroid treatment are used to manage persistent or severe AD cases. Although glucocorticoids exhibit therapeutic efficacy for patients with mild-to-moderate AD, long-term application can induce adverse cutaneous effects, including skin dryness, atrophy, stratum corneum weakening, pigmentation, and superficial capillary dilation. Furthermore, the long-term application of glucocorticoids may impair skin barrier, disrupt surface microbial communities, and exacerbate inflammatory reactions within skin tissue (31). These limitations underscore the persistent clinical need for novel topical therapies with improved safety, efficacy, and manageability.

Fuzhiqing ointment replenishes moisture and lipids, restoring the skin’s natural barrier, reducing transepidermal water loss, and maintaining its physiological acid–base balance (32). Besides, the ointment might modulate the skin microbiome by inhibiting harmful bacteria like *Staphylococcus aureus* and supporting beneficial microbes, potentially reducing inflammation. Meanwhile, its immune-modulatory effects might restore immune homeostasis by downregulating Th2-driven inflammation and promoting regulatory T cell activity (33). These mechanisms suggest that Fuzhiqing ointment could provide a multifaceted approach to AD management, but further clinical studies are needed to confirm its efficacy and underlying mechanisms. In the treatment of skin disease, herbal topical treatments and emerging biologics, such as dupilumab, exhibit distinct efficacy profiles. Herbal remedies are effective for mild to moderate cases, offering gentle symptom relief and improving skin barrier function, though their effects are often slower and less potent. In contrast, biologics target immune pathways, providing rapid and robust improvement in severe cases, but with higher costs and potential side effects (34–36). Therefore, herbal topical treatments, with their effectiveness, high safety profile, and lower cost, still offer significant advantages in the treatment of mild to moderate skin conditions.

Previous studies indicate that Fuzhiqing ointment effectively alleviate neurodermatitis symptoms, improves quality of life, and exhibits a favorable safety profile (32). In addition, clinical study also indicates that the combination of Fuzhiqing ointment and Heparin sodium ointment can improve eczema symptoms by mitigating the release of inflammatory factors and protecting nerve endings, with fewer adverse reactions (33). Furthermore, urea vitamin E cream has been shown to enhance skin hydration, elasticity, and barrier function, with the synergistic effects of urea’s moisture retention and Vitamin E’s antioxidant properties make it widely applicable and well-tolerated across diverse patient populations (37, 38). Therefore, this study design incorporates foundational therapy (urea-vitamin E cream) with rescue medication (cetirizine hydrochloride tablets for refractory pruritus), ensuring all AD participants receive standardized background care throughout the trial. This clinical trial aims to systematically evaluate the therapeutic efficacy of Fuzhiqing ointment in mild-to-moderate AD through multidimensional assessment of core domains: skin lesion severity, pruritus intensity, and quality of life. Study findings will facilitate the adoption of Fuzhiqing ointment in clinical practice as an evidence based therapeutic option for mild-to-moderate AD, offering patients with effective symptom mitigation, reduced disease management expenditure, and optimized sustained disease control.

This study is subject to several limitations. First, participant recruitment is restricted to individuals with mild-to-moderate AD, enhancing internal validity while potentially limiting the generalizability of findings to broader populations. Second, the protocol mandates a 2-week active treatment phase followed by a 4-week observational follow-up period, which limits the observation of long-term effects of Fuzhiqing ointment, as well as its efficacy of diseases recurrence prevention in patients with AD. Third, differences of climate (temperature, humidity, ultraviolet radiation, seasonal changes), lifestyle habits (high-fatty food consumption, high-calorie diets, tobacco smoking, alcohol drinking) and other extrinsic factors (cultural difference, occupational exposure, etc.) in the 10 selected hospitals may cause information bias. Fourth, patients in this study will be instructed not to use any additional treatment for AD which might induce a relatively high rate of withdrawal.
